# Synergistic Effects of Graphene Oxide and Nanocellulose on Water-Based Drilling Fluids: Improved Filtration and Shale Stabilization

**DOI:** 10.3390/polym17070949

**Published:** 2025-03-31

**Authors:** Yerlan Kanatovich Ospanov, Gulzhan Abdullaevna Kudaikulova

**Affiliations:** Department of Petroleum Engineering, Satbayev University, Almaty 050000, Kazakhstan; g.kudaikulova@satbayev.university

**Keywords:** drilling fluids, graphene oxide, nanocellulose, wellbore instability, shale stabilization

## Abstract

Shale formations pose significant challenges to traditional drilling fluids, including issues such as fluid invasion, cutting dispersion, and shale swelling, contributing to wellbore instability. While oil-based drilling fluids (OBM) effectively address these challenges, concerns over their environmental impact and cost limit their widespread adoption. Nanoparticles (NPs) have emerged as a promising frontier for enhancing the performance of water-based drilling fluids (WBDFs) in shale applications. This study examines the effectiveness of water-based drilling fluids (WBDFs) enhanced with a nanocomposite of graphene oxide (GO) and nanocellulose (NC) compared to that of conventional WBDFs. The combination of GO and NC is chosen for its synergistic effects: GO provides enhanced mechanical strength and barrier properties, while NC serves to stabilize the dispersion and improve the compatibility with WBDF matrices. The modification with NC aims to optimize the interaction between GO and the drilling fluid components, enhancing performance in regards to shale inhibition and fluid loss control. This research involved the successful synthesis and characterization of a GO/NC nanocomposite, which underwent examination through FTIR, PSD, and SEM analyses. We also evaluated the filtration properties of water-based drilling fluids (WBDF) enhanced with a graphene oxide/nanocellulose (GO/NC) nanocomposite and compared the results to those for conventional WBDF. Filtration performance was assessed under both low-temperature, low-pressure (LTLP) and high-temperature, high-pressure (HTHP) conditions, and contact angle measurements were conducted to examine the wettability of the shale. The results demonstrated that incorporating GO/NC into the WBDF reduced the filtrate volume by 17% under LTLP conditions and by 23.75% under HTHP conditions, indicating a significant improvement in filtration control. Furthermore, the GO/NC-WBDF increased the hydrophobicity of the shale, as shown by a 61° increase in the contact angle. These findings suggest that GO/NC enhances the performance of WBDF, particularly in unconventional shale formations, by reducing fluid loss and improving wellbore stability.

## 1. Introduction

Throughout drilling operations, shale formations constitute approximately three-quarters of the encountered formations, with shale instability accounting for roughly 90% of wellbore instability issues [1,2]. Despite extensive research on shale instability in recent decades, it remains a significant concern in the petroleum industry [3,4]. Additionally, the emergence of shale gas as an unconventional energy source has transformed the global energy landscape, yet challenges related to shale instability, particularly in horizontal sections, pose significant obstacles to its advancement [5,6]. Shale formations are characterized by minimal porosity and permeability, making it challenging to establish a filter cake on the shale surface using traditional drilling fluid additives, which are often too large to effectively bridge and seal the nanopores within the shale formations [7]. Therefore, careful selection of drilling fluid and additives is crucial to address these challenges. Although oil-based drilling fluids offer excellent performance due to minimal chemical interactions and exceptional stability under downhole conditions, their high cost and adverse environmental impacts present significant drawbacks [8]. As a result, there is a growing preference for water-based drilling fluids (WBM) over oil-based alternatives. However, WBM is prone to interacting with the clays present in shale formations, necessitating the development and assessment of drilling fluids formulated with nanoparticles to enhance their efficacy in such environments. Nanoparticles (NPs), typically ranging in size from 1 to 100 nm, have the capability to physically block shale pores, thereby reducing filtrate invasion and improving wellbore stability [9,10]. Moreover, NPs boast an exceptionally high surface area-to-volume ratio, allowing them to be added at low concentrations [11]. Incorporating NPs offers advantages such as reducing fluid losses and filter cake thickness under both low-temperature, low-pressure (LTLP) and high-temperature, high-pressure (HTHP) conditions. Furthermore, NPs have been shown to enhance the rheological properties of WBM, preventing excessive gel strength [12,13]. While research regarding the utilization of NPs as additives for WBM has surged in recent years, few studies have explored their potential to enhance the inhibition capability of WBM. In previous research, mesoporous nanosilica has been utilized to enhance drilling fluids aimed at improving shale stability, reducing filtrate loss, and minimizing swelling. The incorporation of mesoporous nanosilica demonstrated significant improvements in these areas, showcasing its potential in addressing the challenges associated with drilling operations in shale formations. The high surface area and controlled pore structure of mesoporous nanosilica contributed to its effectiveness in these applications, leading to promising results [14]. Silica nanoparticles coated with AEAPTS ([3-(2-Aminoethylamino) propyl] trimethoxy silane) have been shown to significantly enhance the rheological properties and filtration control of water-based drilling fluids. These advancements highlight the potential of AEAPTS-coated nanoparticle additives for improving drilling fluid performance under challenging high-temperature conditions [15]. Biogenic copper oxide nanoparticles (CuO NPs), synthesized using natural extracts, have shown significant potential in enhancing drilling fluid performance. These nanoparticles have demonstrated improvements in lubricity, filtration, and rheological properties, making them promising additives for high-temperature drilling operations [16]. One promising avenue for improving the performance of WBDFs involves a GO/NC nanocomposite, offering a novel approach for enhancing rheological properties and stabilizing shale formations. While GO has demonstrated efficacy in plugging shale pores effectively, its application is not without limitations. Specifically, at high concentrations, GO can agglomerate, compromising its effectiveness and hindering its dispersion within drilling fluids. To overcome these limitations, researchers have turned to nanocellulose, aiming to capitalize on its unique properties, to address the challenges associated with GO utilization. This research uniquely combines GO with NC to create a composite that addresses the limitations observed with high concentrations of GO alone, such as agglomeration and dispersion challenges. Unlike previous studies that only evaluated individual nanoparticles, our study demonstrates the synergistic effect of GO and NC in enhancing the performance of WBDFs, resulting in superior shale stabilization and improved filtration properties. This novel combination reduced the filtrate volume by 17% under LTLP and 23.75% under HTHP conditions, demonstrating significant improvements in filtration control. Additionally, the GO/NC-WBDF increased shale hydrophobicity, as indicated by a 61° rise in the contact angle. These findings suggest that GO/NC enhances WBDF performance by minimizing fluid loss and improving wellbore stability in unconventional shale formations. While GO/NC composites have been used in other applications, to the best of our knowledge, this composite has not been previously applied in water-based drilling fluids. Our study seeks to fill this gap, focusing on the unique advantages of GO/NC in enhancing fluid performance, specifically in shale formations.

## 2. Materials and Methods

### 2.1. Materials

The study utilized nanocellulose (NC) and graphene oxide (GO), both of which were obtained from Sarsen Amanzholov East Kazakhstan University in Ust-Kamenogorsk, Kazakhstan. These materials were incorporated into the drilling fluid formulation along with conventional additives to enhance its performance. Bentonite was employed as the primary viscosifier, providing the necessary viscosity to suspend drill cuttings during the drilling process. Xanthan gum (XG) was included to further improve the fluid’s rheological properties, ensuring optimal flow behavior under various shear conditions. To control fluid loss, two filtration control agents were added: polyanionic cellulose low-viscosity (PAC-LV) and pre-gelled starch. These components were selected for their ability to minimize filtration into the formation, preserving wellbore stability. Additionally, graphite was used as a lost circulation material (LCM), helping to prevent or mitigate the loss of drilling fluid into fractures or highly permeable formations. Potassium hydroxide (KOH, 85%) was introduced to adjust the fluid’s alkalinity, maintaining the necessary pH level for optimal drilling fluid performance. All additives were sourced from various suppliers and were used in their unmodified, commercially available forms.

### 2.2. Modification of GO with NC

To prepare the GO/NC nanocomposite, a methodical approach was adopted to ensure thorough mixing and uniform distribution of the components. The following steps were performed, each chosen for specific reasons:

1. Combination of GO and NC in a 50:50 ratio: Equal volumes of GO and NC were used to ensure a balanced interaction between the two materials. This ratio was selected based on the need to create a homogeneous composite in which the mechanical and chemical properties of both components contribute equally to the final product.
2. Ultrasound treatment with a UZTA-0.15/22-0 apparatus (Alena, St. Petersburg, Russia): The mixture was subjected to ultrasound treatment using the UZTA-0.15/22-0 apparatus, operating at a frequency of 45 kHz. Ultrasonication was chosen because it effectively disperses the components at a microscopic level by creating cavitation bubbles that break down agglomerates, ensuring a uniform distribution of NC within the GO matrix. This technique enhances the interaction between the materials and improves the final properties of the composite.
3. Duration and temperature of ultrasound treatment: The ultrasonication process was conducted at 25 °C for 30 min. The temperature was kept at 25 °C to maintain stability and prevent the thermal degradation of the nanocellulose, as higher temperatures could lead to unwanted changes in its structure. The 30 min duration was selected based on the methods of previous studies, which indicated this time frame as optimal for achieving uniform dispersion without causing damage to the nanostructures of GO or NC [17].
4. Casting of the solution on a flat plastic surface: After ultrasonication, the well-mixed solution was poured onto a flat plastic surface. This step was critical for creating a uniform thin film. A flat surface allows the liquid to spread evenly, ensuring that the film dries with a consistent thickness, which is essential for achieving reliable mechanical and structural properties in the final material.
5. Drying at room temperature for 48 h: The solution was left to dry at room temperature for 48 h to allow for the slow evaporation of the solvent, leading to gradual film formation. This approach was chosen to avoid the introduction of internal stresses that could arise from rapid drying, which could cause cracks or an uneven thickness. Room temperature drying helps in forming a smooth and continuous film, with a final thickness of 38 μm.
6. Storage in a desiccator: To prevent the absorption of moisture and carbon dioxide from the atmosphere, the resulting film was stored in a desiccator. Nanomaterials like GO and NC are highly sensitive to atmospheric conditions, and exposure to humidity or CO_2_ could affect their physical and chemical properties. Storing the samples in a desiccator ensures long-term stability and preserves the integrity of the material for future testing and analysis.

Each step in this process was carefully chosen to ensure that the GO/NC composite was uniformly mixed, mechanically stable, and chemically consistent, making it suitable for further applications.

### 2.3. FTIR Spectroscopy

FTIR analysis was performed using an FTIR spectrometer, model FT-801 (Simex, Moscow, Russia), following standard procedures. The spectrometer operated with a resolution of 1 cm^−1^, covering a wavelength range from 450 to 4700 cm^−1^. A single-use universal attenuated total reflection (ATR) and mirror-diffuse reflection setup with the upper position model was used to ensure precise measurement. The analysis was conducted at a controlled temperature of 25 °C, with 100 scans captured to ensure accurate and reproducible results.

### 2.4. SEM Analysis

The structure of the GO/NC nanocomposite was investigated using a scanning electron microscope. Surface morphology analysis was performed using the SEM Quanta 200i 3D (FEITM, Eindhoven, The Netherlands). Measurements were conducted under high vacuum conditions, utilizing a secondary electron detector at an accelerating voltage of 15 kV. To enhance electron transfer, the surface of the GO/NC nanocomposite was coated with aluminum. The specimens were mounted on aluminum pins, using carbon tape for stability.

### 2.5. The Particle Size of NC and GO Suspensions

The particle size of the NC and GO suspensions was assessed using dynamic laser light scattering with a Zetasizer NanoZS 90 instrument from Malvern, UK. Aqueous suspensions were subjected to processing at 30 kHz for 10 min using an ultrasonic dispersant U-sonic UZTA-0.15/22-0 from Alena, Russia.

### 2.6. Preparation of WBDFs

This formulation served as the foundation for creating the base fluid, which consisted of water at a concentration of 1 lbm/bbl, bentonite at 10 lbm/bbl, xanthan gum at 0.25 lbm/bbl, starch at 1.85 lbm/bbl, PAC-LV at 1.85 lbm/bbl, and potassium hydroxide (KOH) added to adjust the pH to 9.5. The preparation process began by adding bentonite to deionized water while stirring at 11,000 rpm for 30 min. The resulting slurry was left to hydrate, undisturbed, for 12 h at 77 °F, allowing the bentonite to fully swell and disperse. Once the bentonite was hydrated, the remaining additives were incorporated. “lbm/bbl” is an industry-standard unit referring to “pounds mass per barrel”.

For the nanoparticle dispersion, 300 mL of the base fluid was set aside. GO/NC, at a concentration of 2 wt%, was then introduced into the 300 mL of deionized water and stirred using a magnetic stirrer. To ensure a more uniform and stable dispersion, the mixture underwent an ultrasonication process for one hour at 40 kHz and 185 W. This final step resulted in the preparation of the GO/NC-WBDF.

### 2.7. Drilling Fluid Properties Measurements

#### 2.7.1. Contact Angle Measurement

To measure the contact angle, a specific quantity of pre-hydrated sodium bentonite slurry was prepared, into which GO/NC was added at a concentration of 2 wt%. The mixture was stirred magnetically for at least 24 h to ensure a thorough dispersion of the GO/NC within the bentonite slurry. After mixing, the dispersion was evenly coated onto glass slides and left to air dry. Once dried, a water droplet was carefully placed on the surface of the coated glass slide, and images were captured to observe the interaction between the water and the surface.

The contact angle was measured using a contact angle tester (JC2000D, Xi’an, China) following the static sessile drop method. This technique involves analyzing the angle formed by the water droplet at the solid–liquid interface, providing insight into the wettability of the GO/NC-coated surface.

#### 2.7.2. Filtration Properties

The API low-temperature, low-pressure (LTLP) filtration test was conducted at a temperature of 77 °F and a pressure of 100 psi. Standard filter paper with a pore size of 2.7 μm was utilized, and air served as the pressure source. Conversely, the high-temperature, high-pressure (HTHP) filtration test was performed at 250 °F and 500 psi. An Ofite HTHP filter press system was employed, with CO_2_ utilized as the pressure source and standard filter paper employed. In both tests, the filtrate volume was monitored over a 30 min period, with the final volume reported to the nearest 0.1 mL. However, owing to the HTHP filter press area being half that in the LTLP test, the HTHP filtrate volume was doubled, in accordance with API guidelines. The same adjustment was applied to the spurt volume. Additionally, the thickness of the filter cake was measured using a vernier caliper.

## 3. Results and Discussion

### 3.1. The Particle Size of NC and GO Suspensions

**Figure 1:**
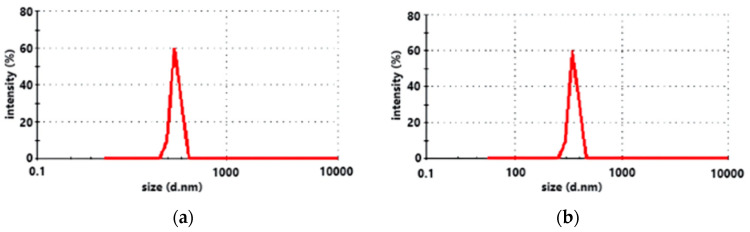
The particle size of the GO (**a**) and NC (**b**) suspensions.

Figure 1 illustrates that the average particle size of GO was 352 nm (Figure 1a), while the average particle size of NC measured 470 nm in length and 80 nm in width (Figure 1b).

### 3.2. FTIR Spectroscopy

**Figure 2:**
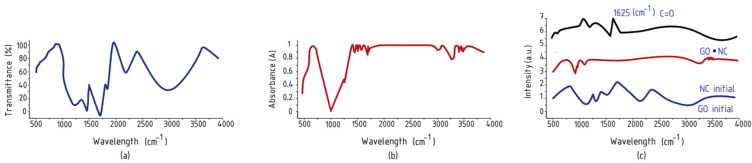
FTIR spectra of GO (**a**); NC (**b**); GO/NC (**c**).

Figure 2a depicts the FTIR spectrum of the initial GO. In this spectrum, a broad peak is evident at 3226 cm^−1^ and another at 1420 cm^−1^, attributed to the stretching vibration mode of the O–H bond. Additionally, the band observed at 1723 cm^−1^ corresponds to the stretching vibration of C=O bonds in the carbonyl and carboxyl groups, while the band at 1585 cm^−1^ corresponds to the stretching vibration and deformation peaks of the aromatic ring C=C bonds. Epoxy functional groups are indicated at 1249 cm^−1^ as the C–O stretching vibration, and at peak 1054 cm^−1^, which is attributed to the stretching vibration of C–O alkoxy bonds [18,19]. Figure 2b presents the IR spectrum of nanocellulose. In this spectrum, the absorbance region at wavenumber 900 cm^−1^ is associated with the deformation of the glycoside C–H bonds. Additionally, absorption peaks observed at 1100 cm^−1^, 1430 cm^−1^, and 2880 cm^−1^ are attributed to the symmetrical and asymmetrical stretching vibration signals of the C–H, CH_2_, and C–H groups, respectively. The broad absorbance band appearing in the range from 3300 cm^−1^ to 3500 cm^−1^ is characterized by the valence stretching vibration of the O–H groups. The findings of this study align well with the results of previous work [17,20,21]. Figure 2c displays the IR spectra of the GO/NC nanocomposite. A notable change in this spectrum is the occurrence of the valence stretching vibration of the ether carboxyl C=O bond in the absorption region at 1625 cm^−1^. This indicates the formation of an etheric O=C–OH bond between the OH group in the cellulose molecule and the carboxyl groups in the GO molecule. These results are consistent with those reported in the literature [18,22].

Table 1 presents the FTIR spectroscopy data for graphene oxide (GO), nanocellulose (NC), and the GO/NC composite, detailing the characteristic peaks and corresponding functional groups for each material.

### 3.3. SEM Analysis

The selection of GO and NC was based on their size, which is theoretically compatible with the pore size of the shale sample. This suggests their potential effectiveness as pore-plugging agents in shale applications, as documented in prior studies [23,24]. Additionally, the incorporation of GO was driven by the expectation that, under downhole conditions, its flexible nature could effectively seal shale fissures, potentially minimizing the formation of microfractures along the bedding planes of the shale. This could, in turn, reduce water infiltration into the shale matrix, thereby improving wellbore stability [25].

**Figure 3:**
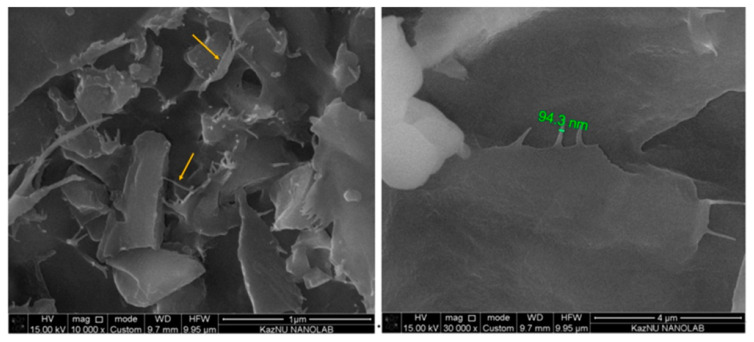
SEM images of GO/NC nanocomposite film.

The surface morphology of the GO/NC nanocomposite film is depicted in Figure 3. SEM images reveal the presence of CNF, which serves as a contact layer between the ultrathin layers of GO. Some of the GO sheets are oriented perpendicular (standing) to the membrane surface, while the majority of the GO layers lie horizontally atop the CNF. These findings are in good agreement with those noted in the previous literature [26].

### 3.4. Contact Angle Measurement

**Figure 4:**
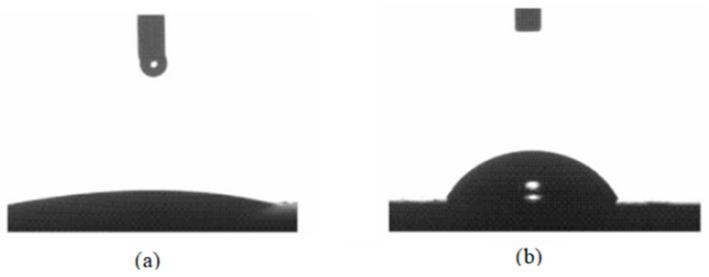
Contact angle measurement of (**a**) bentonite slurry; (**b**) bentonite slurry containing 2 *w*/*v*% GO/NC nanocomposite.

Figure 4 shows the contact angle measurements, highlighting a significant shift in surface wettability. The initial contact angle for the bentonite slurry was 14.3°, demonstrating its strong hydrophilic nature. However, after incorporating 2 wt% graphene oxide/nanocellulose (GO/NC) into the slurry, the contact angle increased to approximately 61°, indicating a marked improvement in hydrophobicity. This increase suggests that the shale surface became less susceptible to water adsorption, which is beneficial for reducing water infiltration. Consequently, the addition of GO/NC enhances shale stability, making it a promising modification for drilling fluid formulations. The modification of graphene oxide (GO) with nanocellulose (NC) was specifically designed to balance hydrophobic and hydrophilic properties. While the addition of NC to GO increases the overall hydrophobicity of the nanocomposite, this modification does not adversely affect the aqueous dispersion stability. The hydrophilic hydroxyl groups present in the nanocellulose maintain sufficient interaction with water molecules, thereby preserving the dispersion stability in an aqueous medium. Furthermore, our experimental results have shown that the modified GO/NC nanocomposite remains well-dispersed in the water-based drilling fluid, which is crucial for its effective application to enhance shale stability. Thus, the hydrophobic modification achieved through the incorporation of nanocellulose does not compromise the dispersion stability of the additives in the water-based drilling fluid.

### 3.5. Filtration Properties

The results demonstrated that incorporating GO/NC into the base fluid resulted in a 17% reduction in low-temperature, low-pressure (LTLP) filtrate loss. Under high-temperature, high-pressure (HTHP) conditions, the GO/NC-WBDF achieved an even greater reduction, decreasing filtrate loss by 23.75%. This highlights the superior ability of GO/NC to enhance filtration control. Additionally, GO/NC-WBDF reduced filter cake thickness by more than 35% in both the LTLP and HTHP filtration tests. This significant reduction in filter cake thickness indicates a direct improvement in the cake’s permeability. The well-dispersed nanoparticles likely formed a more compact and less permeable filter cake, limiting fluid invasion into the formation. This characteristic is particularly beneficial in unconventional shale formations, where GO/NC-WBDF can form an external filter cake on the nanopore structure of the shale, thereby reducing fluid invasion, stabilizing pore pressure, and improving wellbore stability. Previous studies have reported similar findings with graphene oxide (GO), demonstrating the effectiveness of nanoparticle additives in reducing filtrate loss, especially in formations with pore sizes around 3 μm. It is important to note that the addition of nanoparticles also influences the structure and thickness of the filter cake.

**Figure 5:**
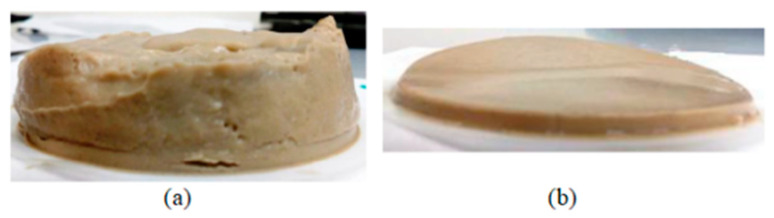
Filter cakes formed by the base WBDF (**a**); GO/NC-WBDF (**b**).

Figure 5a,b compares the filter cakes formed by base fluid and GO/NC-WBDF. In the case of base fluid, the filter cake is a loose mass of clay particles and microparticles, with a thickness of approximately 1–2 cm. In contrast, the filter cake formed by GO/NC-WBDF is dense, uniform, and much thinner, measuring 3–5 mm in thickness, with high adhesion to the filter. This improvement in filter cake structure further supports the enhanced performance of GO/NC-WBDF in reducing fluid loss and maintaining wellbore stability.

The analysis revealed that the nanoparticles effectively bridged and sealed the pore throats and microcracks within the shale cores, forming a dense plugging film on the shale surface and enhancing shale stability during the drilling process. The inhibitory mechanism of GO/NC against shale hydration can be attributed to its small particle size, large specific surface area, and high surface energy, facilitating strong surface activity and easy adsorption onto clay particle surfaces. Additionally, the presence of residual bonds and active hydroxyl groups enhances GO/NC’s adsorption capacity. Furthermore, the surface of GO/NC becomes more hydrophobic, enabling the formation of a hydrophobic film on the shale surface upon contact with the GO/NC solution. This film inhibits clay mineral hydration by restricting water molecule entry into clay particle clearances, thereby demonstrating the effective shale inhibition performance of GO/NC.

### 3.6. Mechanism Analysis

**Figure 6:**
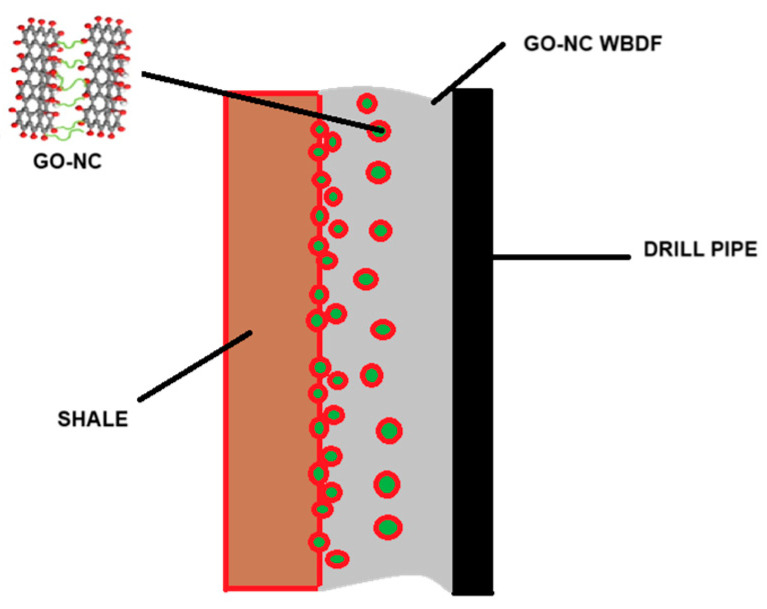
Mechanism analysis of GO/NC-WBDF interaction with shale formation.

The results of this study demonstrate that the GO/NC-WBDF significantly improves wellbore stability compared to that of traditional WBDFs. The improved performance of GO/NC-WBDF can be attributed to the unique interaction between GO and NC. The GO/NC composite effectively bridges and seals the nanopores in the shale, creating a dense plugging film that enhances shale stability. The NC component improves the dispersion of GO, preventing agglomeration and ensuring uniform distribution throughout the fluid. This synergistic effect results in reduced fluid invasion, lower swelling rates, and improved rheological properties, which are all crucial for maintaining wellbore stability during drilling operations. Figure 6 illustrates the mechanism analysis of the GO/NC-WBDF interaction with shale formation.

## 4. Conclusions

This study successfully demonstrated the potential of incorporating graphene oxide/nanocellulose (GO/NC) nanocomposites into water-based drilling fluids (WBDF) to enhance their filtration properties and overall performance. The results indicated that the addition of GO/NC significantly reduced the filtrate volume by 17% under low-temperature, low-pressure (LTLP) conditions and by 23.75% under high-temperature, high-pressure (HTHP) conditions, highlighting its effectiveness in improving filtration control. Furthermore, the GO/NC-WBDF exhibited a notable increase in the hydrophobicity of shale, evidenced by a 61° rise in the contact angle measurements. These findings underscore the synergistic benefits of using GO and NC together, offering enhanced shale stabilization and improved rheological properties compared to the results for conventional WBDF. The application of GO/NC not only promotes more effective fluid loss control but also contributes to improved wellbore stability, making it particularly advantageous when drilling in unconventional shale formations.

In conclusion, the integration of GO/NC into WBDF presents a promising approach for developing more efficient and environmentally friendly drilling fluids, ultimately leading to more sustainable drilling practices in the oil and gas industry. Future research should focus on optimizing the formulation and exploring the long-term performance of GO/NC-enhanced fluids in various drilling environments.

## Figures and Tables

**Table 1:** FTIR spectroscopy for GO, NC, and GO/NC.

Material	Functional Group	Wavenumber cm^−1^	Peak Description
Graphene Oxide (GO)	O–H stretching	3226	Broad peak, O–H bond stretching vibration
	O–H stretching	1420	O-H bond stretching vibration
	C=O stretching	1723	Carbonyl and carboxyl group C=O bond stretching vibration
	C=C stretching/deformation	1585	Aromatic ring C=C bonds
	C–O stretching (epoxy)	1249	Epoxy functional groups
	C–O stretching (alkoxy)	1054	Alkoxy bond stretching
Nanocellulose (NC)	C–H deformation	900	Glycoside bond deformation
	C–H Symmetric/Assymetric	1100	Symmetric/Assymetric stretching of C–H, CH_2_, C–H groups
	CH_2_ Symmetric/Assymetric	1430	Symmetric/Assymetric stretching of C–H, CH_2_, C–H groups
	C–H Symmetric/Assymetric	2880	Symmetric/Assymetric stretching of C–H, CH_2_, C–H groups
	O–H stretching	3300–3500	Valence stretching of O–H groups
GO/NC Nanocomposite	C=O (ether carboxyl)	1625	Etheric O=C–OH bond between OH in NC and carboxyl in GO

## Data Availability

The original contributions presented in the study are included in the article, further inquiries can be directed to the corresponding author.
